# *P*-hydroxybenzaldehyde protects *Caenorhabditis elegans* from oxidative stress and *β*-amyloid toxicity

**DOI:** 10.3389/fnagi.2024.1414956

**Published:** 2024-05-22

**Authors:** Xingzhi Yu, Jie Tao, Tian Xiao, Xiaohua Duan

**Affiliations:** Yunnan Key Laboratory of Dai and Yi Medicines, Yunnan University of Chinese Medicine, Kunming, Yunnan, China

**Keywords:** *p*-hydroxybenzaldehyde, Alzheimer’s disease, *Caenorhabditis elegans*, neuroprotection, oxidative stress, Aβ protein

## Abstract

**Introduction:**

*Gastrodia elata* is the dried tuber of the orchid *Gastrodia elata* Bl. It is considered a food consisting of a source of precious medicinal herbs, whose chemical composition is relatively rich. *Gastrodia elata* and its extracted fractions have been shown to have neuroprotective effects. *P*-hydroxybenzaldehyde (*p*-HBA), as one of the main active components of *Gastrodia elata*, has anti-inflammatory, antioxidative stress, and cerebral protective effects, which has potential for the treatment of Alzheimer’s disease (AD). The aim of this study was to verify the role of *p*-HBA in AD treatment and to investigate its mechanism of action in depth based using the *Caenorhabditis elegans* (*C. elegans*) model.

**Methods:**

In this study, we used paralysis, lifespan, behavioral and antistress experiments to investigate the effects of *p*-HBA on AD and aging. Furthermore, we performed reactive oxygen species (ROS) assay, thioflavin S staining, RNA-seq analysis, qPCR validation, PCR Array, and GFP reporter gene worm experiment to determine the anti-AD effects of *p*-HBA, as well as in-depth studies on its mechanisms.

**Results:**

*p*-HBA was able to delay paralysis, improve mobility and resistance to stress, and delay aging in the AD nematode model. Further mechanistic studies showed that ROS and lipofuscin levels, Aβ aggregation, and toxicity were reduced after *p*-HBA treatment, suggesting that *p*-HBA ameliorated Aβ-induced toxicity by enhancing antioxidant and anti-aging activity and inhibiting Aβ aggregation. *p*-HBA had a therapeutic effect on AD by improving stress resistance, as indicated by the down-regulation of NLP-29 and UCR-11 expression and up-regulation of PQN-75 and LYS-3 expression. In addition, the gene microarray showed that *p*-HBA treatment played a positive role in genes related to AD, anti-aging, ribosomal protein pathway, and glucose metabolism, which were collectively involved in the anti-AD mechanism of *p*-HBA. Finally, we also found that *p*-HBA promoted nuclear localization of DAF-16 and increased the expression of SKN-1, SOD-3, and GST-4, which contributed significantly to inhibition of Aβ toxicity and enhancement of antioxidative stress.

**Conclusion:**

Our work suggests that *p*-HBA has some antioxidant and anti-aging activities. It may be a viable candidate for the treatment and prevention of Alzheimer’s disease.

## Graphical abstract

In this study, based on the *C. elegans* AD model, the optimal concentration of *p*-hydroxybenzaldehyde (*p*-HBA) was determined to be 0.5 mM through a paralysis experiment, and this concentration was used in subsequent studies. First, anti-AD efficacy was evaluated, including lifespan, anti-stress, and ROS measurements, etc. Then, transcriptomic sequencing analysis, PCR verification and gene chip determination were performed to further investigate the anti-AD mechanism of *p*-HBA. Finally, to clarify the molecular response of the protective effect of *p*-HBA on *Caenorhabditis elegans*, we combined with the GFP reporter gene worm experiment to detect the expression of 4 genes related to aging and antioxidant in worms. (By Figdraw: http://www.figdraw.com).

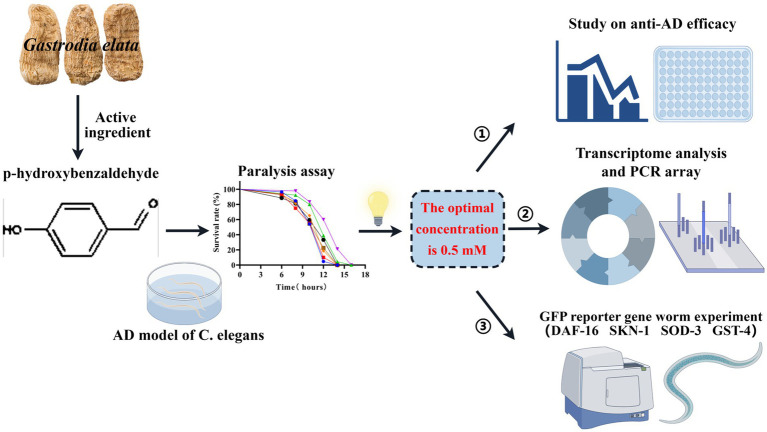

## 1 Introduction

Alzheimer’s disease (AD), also known as senile dementia, is a neurodegenerative disease closely related to aging with clinical manifestations of progressive memory loss or even cognitive dysfunction, and an inability to take care of oneself, which is more common in the older population (Kandimalla and Reddy, 2017). Its two main pathological hallmarks are the accumulation of extracellular aggregates of amyloid *β*-protein (Aβ) (in the form of amyloid plaques) and highly phosphorylated intracellular aggregates (in the form of neurofibrillary tangles) (Cerasuolo et al., 2023). Amyloid precursor protein (APP) is a transmembrane protein present in neurons and glial cells of the brain. Under pathological conditions, APP is cleaved in the amyloid pathway via *β*-secretase to form APP-β, which is then cleaved by *γ*-secretase to produce the Aβ40/42 monomer fragment, which is abnormally aggregated in the extracellular environment of Aβ and induces fibrosis leading to the formation of age spots. On the one hand, Aβ aggregation can produce direct toxic effects on neuronal cells; on the other hand, oligomers formed by Aβ aggregation can stimulate inflammation and the activation of the oxidative stress cascade. At the same time, oxidative stress also acts on mitochondria, affecting energy metabolism, accelerating tau protein phosphorylation, and exerting neurotoxicity through the formation of neurofibrillary tangles and lowering excitability thresholds, thus accelerating damage to neuronal cells (Kam et al., 2013). The onset of AD is insidious, and the course of the disease is relatively long. The cognitive and memory dysfunctions and low motor and behavioral abilities that occur have a serious impact on the quality of life of patients.

According to the findings of the 2018 World Alzheimer’s Disease Report, there is a case of AD diagnosed every 3 s worldwide (Liu J. Y. et al., 2023). Compared to the 5.5 million cases in 2018, the number of patients with AD who are 65 and older in developed countries will reach 7.1 million by 2025, an increase of nearly 29%. Unless there is a major medical breakthrough, the number of people with AD is likely to nearly triple to 13.8 million by 2050, a dramatic increase in prevalence that has attracted global attention (Zhu et al., 2022). However, there are few internationally recognized drugs that can be used to treat AD. In the clinic, AD is treated mainly with cholinesterase inhibitors such as donepezil and galantamine, as well as *N*-methyl-d-aspartate (NMDA) receptor antagonists such as amantadine, which are all focused on symptomatic improvement and lack efficacy to improve the course of the disease (Tan W. et al., 2022). Today, researchers are gradually shifting the research and development of anti-AD drugs toward the study of the efficacy of natural products. Studies have shown that the chemical nature and structural complexity of natural products are of great potential value in drug development (Zhang et al., 2023) and have gradually become an important source of new lead compounds in drug discovery and development, allowing more and more structures and properties of natural products to be confirmed (Newman and Cragg, 2020). These natural products have also made great progress in the research and application of anti-AD and other neurodegenerative diseases. For example, studies have shown that the ginsenosides Rd., Rg2 and Rh1 all have neuroprotective effects, and Rd. can reduce the neurotoxic effects of Aβ protein on *C. elegans* by resisting oxidative stress (Mi et al., 2022), while ginsenosides Rg2 and Rh1 also have strong pharmacological activities in the nervous system, and can improve the body’s ability to resist neuronal damage, regulate neural activity, and can treat AD and vascular dementia (Liu S. et al., 2023). Furthermore, Frondoside A, a saponin isolated from sea cucumber, can reduce the level of ROS in nematodes, decrease the oligomerization of Aβ proteins, and inhibit their toxicity (Tangrodchanapong et al., 2020). The neuroprotective properties of parthenolide include the reduction of oxidative stress by regulating the AMPK/GSK3β (ser9)/Nrf2 signaling pathway, thus inhibiting the apoptosis cascade, and playing a role in reducing the toxicity of Aβ proteins (Sun et al., 2023). Thus, the expansion of research into natural product research has been of great importance for the development of new anti-AD drugs.

Neurodegenerative diseases, including AD, are a major and growing burden around the world, and the validation of factors that reduce their impact is an urgent challenge that also requires a great deal of research (Zhao et al., 2023). There are relatively more animal models available to study AD today, such as APP/PS1 double transgenic mice, zebrafish, Drosophila. Nonetheless, such models are not conducive to high-throughput screening of drugs due to culture period, experimental consumption, and holistic evaluation, whereas the *C. elegans* model has received extensive attention in drug screening for AD due to its own unique advantages. *C. elegans* is an easy-to-operate and multifunctional model organism that simplifies the complexity in drug discovery and drug target identification (Wang and Zheng, 2022). Its adult nematodes are approximately 1 mm long, tiny, and transparent, allowing tracking of neuronal structure and morphology through the expression of green fluorescent protein (GFP) in specific neurons and visualization of cellular location using GFP-tagged proteins (Chalfie et al., 1994). Approximately 83% of the *C. elegans* proteome is predicted to have human homologs (Lai et al., 2000), and approximately 50% of *C. elegans* protein-coding genes have functional homologs in humans (Kim et al., 2018). As a result, there is a high degree of homology between *C. elegans* and human genes involved in processes such as aging, apoptosis, cell signaling, metabolism, or the cell cycle; thus, *C. elegans* can be used to study many important molecular pathways in complex organisms (Guarente and Kenyon, 2000; Tissenbaum, 2015). Currently, by modern scientific and technological means, a variety of AD-associated *C. elegans* models have been successfully developed, which mimic pathological characteristics such as Aβ deposition in organisms. Combined with the advantages of a short life cycle of nematodes, a clear genetic background, and high genetic homology, it makes it easier and faster for researchers to screen drugs and explore the association and mechanisms involved in diseases and drug activity. In addition, studies have confirmed the potential advantages of *C. elegans* as an AD model to study the efficacy of natural products. Flavonol glycoside complanatoside A has been shown to enhance resistance of *C. elegans* by activating various cytoprotective pathways such as FOXO/DAF-16, NRF2/SCN-1, and HSF-1, which exert antioxidant and anti-aging activities to delay neurodegenerative diseases (Tan L. et al., 2022). Scorpion venom heat resistant peptide (SVHRP), which is isolated from scorpion venom, not only attenuates paralysis and chemotaxis dysfunction in transgenic *C. elegans* expressing Aβ protein, but also regulates Aβ oligomers and attenuates ROS levels in transgenic *C. elegans* (Zhang et al., 2016). The oolong tea polyphenols OFA and OFB not only delay Aβ-induced paralysis in transgenic strain CL4176 and counteract the chemotaxis defect of strain CL2355 but also prolonged the lifespan of *C. elegans*, and can be expected to be a promising candidate for the future treatment of age-related neurodegeneration (Zhang et al., 2022). In summary, the use of *C. elegans* as an *in vivo* model for drug screening studies in neurodegenerative disorders, such as AD, is an effective, convenient, and advanced approach (Alexander et al., 2014).

Traditional Chinese medicine (TCM) and ethnomedicine, as well as an important source for the excavation of effective natural products, play an indispensable role in the prevention and treatment of AD. In recent years, research on the anti-AD activity of effective monomer components in natural medicines has become more and more in-depth. Of these, the effective anti-AD components mainly include saponins, phenols, flavonoids, peptides, and alkaloids. Most can effectively improve the inflammatory activity, reduce Aβ plaque deposition as well as its toxic effects, so as to exert neuroprotective effects against AD (Su et al., 2014). Yi nationality’s medicine *Gastrodia elata* (*Gastrodia elata Blume.*), also known as rhizoma gastrodiae, Ding Feng Grass, and Du Yao Zhi, is a perennial herb belonging to the Orchidaceous family, which is suitable for growing in a light-sheltered, well-ventilated and well-watered acidic natural environment (Qiuying and Shunxing, 2001). It has a unique flavor, with the effects of suppressing liver-yang, expelling wind and clearing collaterals, calming wind and stopping spasm, and is mainly used for the treatment of headache and dizziness, numbness of the limbs, hand and foot paralysis and rheumatism paralysis pains (Committee, 1999, 2020; Lili et al., 2023). Moreover, a class of prescriptions containing *Gastrodia elata* is often used in the treatment of cardiovascular and cerebrovascular diseases and aging-related diseases, such as Tianma (*Gastrodia elata*) Gouteng (*Gambir Plant*) Decoction, Tianma Pill, and Banxia (*Pinellia*) Baizhu (*Macrocephalae rhizoma*) Tianma Decoction, which are famous traditional Chinese medicine formulas (Deng et al., 2020; Xu et al., 2022). Modern pharmacological studies have found that it has the effect of increasing intelligence, brain health, neuroprotection, and delaying aging. Moreover, compared with traditional Chinese medicines such as *Polygonatum* and *Deer Antler*, which also have the functions of anti-aging and memory enhancement, *Gastrodia gastrodia’s* effects on memory enhancement, learning ability, and headache resistance are more significant and distinctive and specialized, and its use is more common (Liu et al., 2018; Gong et al., 2024). And in recent years it has also been used in the treatment of cognitive dysfunction, AD, and other related conditions (Gong et al., 2024). Furthermore, some studies have shown that *Gastrodia elata* ethyl acetate extract (EEGE) has certain neuroprotective and cerebral protective effects (Jin-long et al., 2013; Xiao-hua et al., 2013). In our previous study, we found that the EEGE site had the best anti-AD activity of *Gastrodia elata*, and had good *ex vivo* and *in vivo* antioxidant activity, as well as anti-Aβ toxicity, which could improve the symptoms of the *C. elegans* AD model. The drug could modulate the insulin pathway of *Caenorhabditis elegans*, indicating that it could be used in the treatment of neurodegenerative disorders, such as AD (Shi X. et al., 2023). Meanwhile, we isolated *p*-hydroxy benzaldehyde (*p*-HBA), the main active ingredient with a relatively high content, from the EEGE site, as one of the main phenolic components of *Gastrodia elata*, which has been shown in modern studies to have neurorestorative effect on the peri-infarct cortical microenvironment after cerebral ischemia and reperfusion in rats (Yuan et al., 2022). In our previous study, we found that *p*-HBA had a delaying effect on the level of paralysis in the AD *C. elegans* model; thus, we hypothesized that this active ingredient could reduce Aβ clustering and its toxic effects, which has some potential for the prevention and treatment of AD.

In this study, the potential of *p*-HBA, the main phenolic component of EEGE, was investigated for the treatment of AD with regards to its effects on growth and development, motor behavior, anti-aging, and anti-stress activity using the using the *C. elegans* model. In addition, ROS levels, thioflavin S staining of Aβ protein, RNA-seq analysis, qPCR validation, PCR Array, and GFP reporter gene worm experiment were conducted to determine the potential mechanisms of *p*-HBA against AD and to provide a basis and reference for the basic research and development of *Gastrodia elata* pharmacodynamic constituents for the prevention and treatment of AD diseases.

## 2 Materials and methods

### 2.1 Drug preparation, *C. elegans* strains, and maintenance

*P*-hydroxybenzaldehyde (*p*-HBA, AB2539-0020) was purchased from Chengdu Alfa Biotechnology Co., Ltd. *p*-HBA was weighed using the ME104E precision electronic balance, dissolved with deionized water (with 0.2% DMSO as co-solvent) to 10 mM, filtered and sterilized, and then stored at −20°C as the stock solution. The OP50 bacterial solution (OD600 = 0.6) and the stock solution of *p*-HBA were used to prepare different concentrations for drug delivery (0.125, 0.25, 0.5, 1, 2, and 4 mM), which were added to NGM medium. The Control group received bacterial solution in the same volume as that of the drug delivery group, and the optimal concentration of the drug was defined by the paralysis experiments, and then used in all subsequent experiments.

The strains used in this study were transgenic *C. elegans* CL4176 (dvIs27 [myo-3p:A-Beta (1–42):let-851 3’UTR + rol-6(su1006)]X), CL2006 (dvIs2 [pCL12(unc-54/human Abeta(1–42) minige-ne) + rol-6(su1006)]), TJ356 (zIs356[daf-16p:daf-16a/b:GFP + rol-6(su1006)]), LD1 (ldIs7 [skn-1b/c:GFP + rol-6(su1006)]), CF1553 (mu1s84 [pAD76(sod-3:GFP) + rol-6(su1006)]), CL2166 (dvIs19 [(pAF15)gst-4p:GFP:NLS] III), and the wild-type *C. elegans* N2 (wild type). All *C. elegans* strains were purchased from the *C. elegans* Genetics Center (CGC, University of Minnesota, Minneapolis, Minnesota) and fed an uracil synthesis-deficient strain of *Escherichia coli* OP50*. C. elegans* were cultured in solid medium containing *E. coli* OP50 (NGM; consisting of 400 mL of deionized water, which included 1.0 g peptone, LP0042B, Beijing BioDee Biotechnology Co., Ltd.; 1.2 g NaCl, 7.0 g agar, A8190, Beijing Solarbio Science & Technology Co., Ltd.; autoclaved and supplemented with 10 mL of phosphate buffer, 400 μL of 1 mM MgSO_4_, 1 mM CaCl_2_, 400 μL of 5 mg/mL cholesterol solution, 57–88-5, Shanghai Macklin Biochemical Co., Ltd.). All *C. elegans* were stored at 20°C, except CL4176, which was stored at 16°C.

### 2.2 Paralysis assays

CL4176 worms, a temperature-sensitive transgenic AD model, is normally cultured at 16°C and has a curved (C-shaped) body and can move normally in the medium. When the temperature increases to 25°C, the serine/threonine protein kinase 1 system is inactivated, leading to overexpression of Aβ-toxic proteins in the muscle and thus inducing a paralyzed phenotype (Link et al., 2003; Wu et al., 2006). Paralysis was defined as rigid movement or only head movement of the *C. elegans* when its body was mechanically stimulated. The CL4176 worms synchronized with stage L1 were transferred to 35 mm culture plates with or without drugs and incubated at 16°C for approximately 48 h. The temperature was then raised to 25°C for induction of the transgenic paralysis phenotype and the incubation continued for approximately 24 h. After incubation, worms were observed at 2-h intervals until all worms were paralyzed.

### 2.3 Lifespan assays

The CL4176 worms synchronized at stage L4 were transferred to NGM culture plates with or without drug (0.5 mM), 3 plates per group, with a total of not less than 70 worms, and placed at 16°C, at which point they were recorded as worm survival on day 0. To prevent the influence of egg and larval development on worm count, oviposition was inhibited by adding 12 mM floxuridine (FUDR, 50–91–9, Shanghai Macklin Biochemical Co., Ltd.) to the NGM medium. The number of surviving worms was observed and recorded every 2 days until all the worms died.

Worm death and exclusion criteria: worms were considered dead when there was no response to stimulation of the worms’ circumference with a picker and no hyaline ring around the head, and worms that developed into bag-like worms and did not die within the range of coated with the experimental drug were excluded from the statistical data. The experiment was repeated three times independently.

### 2.4 Lipofuscin assays

Worms synchronized to the L4 stage were transferred to NGM plates with or without drug (0.5 mM). After 10 days of incubation at 20°C, worms were collected in PBS buffer and anesthetized by adding levamisole (0.2%, Sichuan Weikeqi-Biotech Co. Ltd., China), and lipofuscin autofluorescence of each group of worms was analyzed under a confocal microscope (LSM900, Carl Zeiss, Germany). Fluorescence intensity values were measured using Image J software. There were at least 20 worms per group. The experiment was repeated three times independently.

### 2.5 Pharyngeal pump beating and motility assays

Pharyngeal pump beating: The CL4176 worms synchronized to stage L3 were transferred to NGM culture plates with or without drug (0.5 mM), with no fewer than 30 worms per group of 3 plates, and placed at 16°C for incubation, at which time it was recorded as day 0. The pharyngeal pumping rate of worms in 30 s was observed under a stereomicroscope from culture to day 4, 6, and 8. Before observation, worms were transferred to new culture plates and 10 were randomly counted in each plate. The worms pharyngeal pump performed one beat upward and downward, respectively, to complete one pumping movement.

Motility: The preculture of CL4176 worms was the same as the pharyngeal pump beating experiment. The motility of the worms was observed on days 4, 6, and 8. Before each observation, the worms were transferred to a new culture plate with 0.1 mL of M9 solution and the number of sinusoidal motions completed within 30 s was counted as an indicator of the motility of the worms, with 10 worms randomly counted in each plate.

### 2.6 Spawning and length assays

Spawning: The total number of worm progeny was used to study the effect of drugs on the reproductive spawning of worms (Chen et al., 2023). The CL4176 worms that were synchronized to stage L4 and had not yet spawned were transferred to NGM culture plates with or without drug (0.5 mM), three plates per group, two worms per plate and placed at 16°C, which was recorded as the first day of worm reproduction, and thereafter the worms were transferred to a new plate every day until the worms no longer spawned. The reproduction of the worms was investigated by counting the number of offspring after incubating plates containing only eggs at 16°C for 48 h.

Body length: N2 worms synchronized to L4 stage were transferred to NGM plates (12 μM FUDR) with or without drug (0.5 mM), and the size of the worms was determined after 2 days of continued incubation. Worms were collected with PBS buffer and anesthetized by adding levamisole (0.2%, Sichuan Weikeqi-biotech Co. Ltd., China), and at least 20 worms in each group were imaged under an inverted biomicroscope [DMI1, Leica Microsystems (Shanghai) Co., Ltd.], and the images were stored. Worm body length (μm) was measured using Image J software. The experiments were repeated three times independently.

### 2.7 Stress tolerance assays

Anti-heat stress: N2 worms synchronized with the L1 stage were transferred to NGM plates with or without drug (0.5 mM), 3 plates per group, with a total of no less than 70 young adult worms, and were incubated at 20°C for 2 d. After incubation, the temperature was raised to 37°C and the survival of the worms was observed and recorded once at 1 h intervals until all the worms were dead. The death of the worm was determined by the absence of movement and swallowing, as well as the absence of response to contact by the platinum wire. The experiment was repeated three times independently.

Antioxidative stress (oxidative stress induced by Juglone): N2 worms synchronized to the L1 stage were transferred to NGM culture plates with or without drug (0.5 mM), and after incubation at 20°C for 2.5 days, the worms were transferred to NGM plates containing Juglone (300 μM, 481–39-0, Shanghai Yuanye Bio-Technology Co., Ltd.), with three plates in each group, and about 30 worms per plate. Subsequently, the survival of the worms was observed every 1 h until all died. Worm death criteria were worms with stiff and motionless bodies that did not respond to light sources and slight vibrations were recorded as dead.

### 2.8 ROS assays

N2 worms synchronized to the L1 stage were transferred to NGM plates with or without drug (0.5 mM), approximately 50 per plate, and incubated at 20°C for 2.5 days. Then, a 5 mM paraquat solution was added to the NGM plates, and after 4 h of treatment, the worms were collected in PBS buffer and washed 3 times. Next, 50 μL of 10 μM DCFH-DA solution (Reactive oxygen species detection kit, S0033S, Beyotime Biotech. Inc.) was added and the worms were incubated at 37°C for 30 min, and washed with PBS 3 times. Next, 30 μL of the worm solution was retrieved and placed on slides, covered by coverslips, and observed under the orthogonal fluorescence microscope (Axio Scope.A1, Carl Zeiss, Inc., United States). The parameters were adjusted and using the saved the images, the fluorescence intensity values were measured using ImageJ software.

### 2.9 Aβ protein staining

Transgenic CL2006 worms synchronized to the L1 stage were transferred to drug-free NGM plates and incubated at 20°C for 2.5 days. After incubation, they were transferred to plates with or without drug (0.5 mM), with approximately 50 per plate. After 2 days of incubation, the worms were collected with M9 buffer and fixed in 4% paraformaldehyde solution (pH 7.4, BL539A, Labgic Technology Co., Ltd.) at 4°C for 24 h. The worms were then treated with permeabilizing solutions [5% *β*-hydrophobic ethanol (60–24-2, Shanghai Macklin Biochemical Co., Ltd.), 1% Triton X-100 (9002-93-1, Meilunbio Biotechnology Co., Ltd.) and 125 mM Tris (77–86-1, pH 7.4, Beijing Solarbio Science & Technology Co., Ltd.)] and were incubated at 37°C for 24 h. The worms were rinsed three times with TBST solution and 100 μL of the fluorescent dye thioflavin S (0.125%, 1,326-12-1, Shanghai Yuanye Bio-Technology Co., Ltd.) was added to the worms, stained for 2 min at room temperature and finally discolored twice with 50% ethanol. CL2006 worms were photographed under a laser confocal microscope (LSM900, Carl Zeiss, Germany): the Aβ protein deposition on the part of the worms before the pharyngeal pump was observed and quantified. Wild-type N2 worms were used as a negative control for the transgenic CL2006 strain.

### 2.10 RNA-seq analysis and real-time PCR validation

Sample preparation: CL4176 worms synchronized at stage L1 were transferred to NGM plates with or without drug (0.5 mM), no less than 1,000 per plate, 5 parallel plates were collected separately for each group, and incubated at 16°C for 48 h, then warmed to 25°C and continued incubating for approximately 30 h. The worms were collected in EP tubes with M9 buffer and washed with sterile water 2–3 times. After snap-freezing with liquid nitrogen, they were stored in an ultra-low-temperature refrigerator at −80°C until sequencing. The gene expression of the transgenic CL4176 worms treated with *p*-HBA and the controls of the untreated CL4176 worms was analyzed using RNA-seq. Total RNA extraction and purification, library construction, and sequencing were obtained from Wuhan Metware Biotechnology Co., Ltd. Next, we filter the downlinked data to get Clean Data. and then sequence comparison with the specified reference genome to get Mapped Data. followed by structural level analyses such as variable splicing analysis, new gene discovery and gene structure optimization. According to the expression amount of genes in different samples or different sample groups, differential expression analysis, differential expression gene function annotation and function enrichment are performed at the expression level. The screening conditions for differential genes were|log2Fold Change| ≥ 1 and FDR <0.05 (Yang et al., 2023).

Real-time fluorescence quantitative polymerase chain reaction (RT-qPCR): sample processing and collection were consistent with the preparation of transcriptome samples. RNA was extracted according to the instructions of the Total RNA Extraction Kit of the AllStyle Gold TransZol Up Plus RNA Kit (ER501-01-V2, TransGen Biotech Co., Ltd.), RNA samples were tested for concentration and purity, cDNA was synthesized using the cDNA reverse transcription kit (A230-10, Beijing GenStar Co., Ltd.), and primers were designed online using Primer3Plus^1^, which were synthesized on behalf of Beijing Tsingke Biotech Co., Ltd. The primer sequences are shown in Table 1. qRT-PCR was carried out using c DNA as template under the following conditions: 95°C for 2 min, 40× (95°C for 15 s, 60°C for 30 s), and 72°C for 30 s. Actin-1 was selected as the internal reference control and the mRNA expression of each gene was calculated using the 2^-△△Ct^ method, which was repeated three times in each group.

**Table 1:** Primer sequences.

Gene	Forward primer (5′–3′)	Reverse primer (5′–3′)
Actin-1	CCACGAGACTTCTTACAACTCCATC	CTTCATGGTTGATGGGGCAAGAG
Nlp-29	CTTGTTCTTGTCGTCCTTCTCG	TTACTTTCCCCATCCTCCATACA
Pqn-75	CACCACGTCAAAAGCGTCAG	AATCCCTTCTTTGGTGGGGC
Lys-3	CGAAACTCCCCAACTCATGC	GCCTGTCTCCATGGTCCAAA
Ucr-11	CGCCTTCACCAACCCTAATTACT	GCTTGTTCCAGAGTGGGATGTA

### 2.11 PCR array

The PCR array, also known as the Gene Function Classification Microarray, is a 96-well plate or 384-well as a carrier, with primers for target genes immobilized in the wells, which can quantitatively and qualitatively detect multiple genes at the same time, with high sensitivity and specificity, and is the first choice for analyzing the expression status of genes related to a signaling pathway or a certain biological function. The processing and collection of the samples were consistent with the preparation of the transcriptome samples. The *C. elegans* RNA was extracted from the blank group and the administration group and reverse transcribed into c DNA. The cDNA stock solution internal reference range: the recommended CT value of ACTB or GAPDH is approximately 18. The mix was diluted and the reverse transcription cDNA (which had been quality checked) was diluted to a total volume of 100 μL and placed aside on ice. The plates were centrifuged at 2000 rpm for 20 s before use, and carefully remove the plate sealing membrane was carefully removed at the end of centrifugation. A reagent mix was prepared as follows: 920 μL of cDNA and qPCR mix (A301-05, Beijing GenStar Co., Ltd.) according to Supplementary Figure>Supplementary Table S1, the prepared mix was shaken and added to the plates at 9 μL per well. The plates were sealed with a transparent sealing membrane after addition. The plates were centrifuged s at 2,000 rpm for 20 s and then tested on the Real-time PCR instrument. The reaction system was set at 10 μL, and the program was: 95°C for 2 min, 40× (95°C for 15 s, 60°C for 30 s) and the and the results were exported for data analysis.

### 2.12 Expression of DAF-16/FOXO, SKN-1/NRF2, GST-4, and SOD-3 in transgenic strains harboring green fluorescent protein (GFP) reporter genes

For all gene expression experiments with the GFP reporter strain, L1 stage worms were incubated on NGM plates with or without drug for 72 h. The animals were then moved to slides and fixed with levamisole anesthesia (2%, 16,595–80-5, Sichuan Vicky Biotechnology Co., Ltd.). Worm images were captured under a Zeiss LSM900 laser confocal microscope (Carl Zeiss, Germany), and photographs were taken at a 10/40x magnification. Image J software was used to analyze the images and subtract the background signal from all readings. For the TJ356 strain, worm plates that were cultured in blank medium for 72 h and then warmed to 37°C for 30 min of induction were used as positive controls.SKN-1::GFP fluorescence intensities were measured in the intestinal region of LD1 worms under the pharynx, and the nucleus localization of DAF-16 was expressed as the number of fluorescence points per worm (Kang et al., 2022). Furthermore, whole-body worms were measured using Image J software to analyze the fluorescence intensity associated with the expression of SOD-3::GFP and GST-4::GFP in CF1553 and CL2166 worms, and the experiments were independently replicated three times, with a minimum of 10 worms in each group for each experiment.

### 2.13 Statistical analyses

The above experiments were repeated independently three times and the experimental data was statistically analyzed and graphed using GraphPad Prism v.8.0, and the experimental results were expressed as mean ± standard deviation. Differences between groups were analyzed by *t*-test or one-way ANOVA, and survival analysis was performed by log-rank test. ^*^p < 0.05, ^**^p < 0.01, ^***^p < 0.001 were considered statistically significant.

## 3 Results

### 3.1 Paralysis experiments to determine the optimal concentration of *p*-HBA

**Figure 1:**
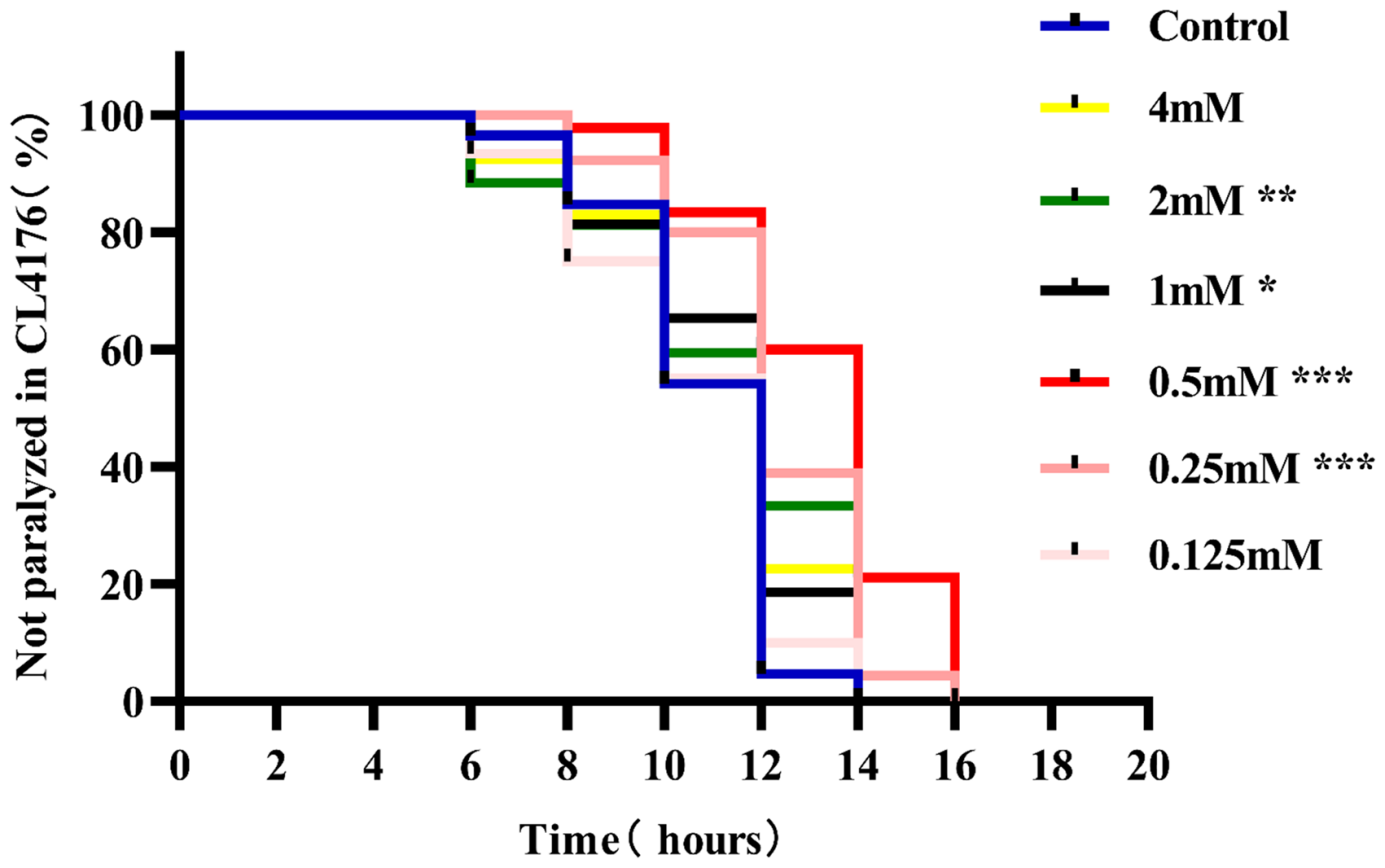
Effect of *p*-HBA on the paralyzed phenotype of AD transgenic CL4176 worms. Synchronized CL4176 worms were treated with different concentrations of *p*-HBA, respectively, and an equal volume of OP50 bacterial solution without drug was used as a blank control (Control group), which was incubated at 16°C for 48 h and then transferred to 25°C to induce the paralytic phenotype. *p*-HBA treatment had a positive effect on delaying worm paralysis. ^*^p < 0.05, ^**^p < 0.01, ^***^p < 0.001.

Paralysis is a prominent phenotype of AD-transgenic CL4176 worms when induced by temperature. When the temperature is increased, Aβ proteotoxicity is expressed in large amounts in muscle cells of the body of the worms, which in turn leads to paralysis of the worms, reflecting the direct toxic effect of Aβ protein aggregation in muscle cells (Alvarez et al., 2022). The effect of different concentrations (0.125, 0.25, 0.5, 1, 2, and 4 mM) of *p*-hydroxybenzaldehyde on the Aβ proteotoxicity of A of transgenic worm strain CL4176 was investigated. None of these concentrations was lethally toxic to CL4176 worms and that all, except 0.125 and 4 mM, prolonged the paralysis time of the worms in the *p*-HBA group compared to the Control group (Figure 1). All *p*-HBA concentrations except 0.125 mM prolonged the worm half paralysis time (PT50, Table 2), of which 0.5 mM prolonged the nematode half paralysis time the longest, and the difference in the overall paralysis rate of delayed nematodes was the most significant (*p* < 0.001), so the optimal concentration of *p*-hydroxybenzaldehyde was 0.5 mM. These results suggest that *p*-hydroxybenzaldehyde has a protective effect on CL4176 Aβ protein-induced toxicity has a protective effect.

**Table 2:** Time to half paralysis for each concentration of drug (*n* = 3).

Drug concentration (*p*-HBA; mM)	Half of the paralyzed time (PT50; h)
0.125	9.311
0	9.430
4	9.627
1	9.725
2	9.812
0.25	11.131
0.5	11.851

### 3.2 *p*-HBA extends lifespan and improves mobility of AD worms, with no effect on their growth and development

**Figure 2:**
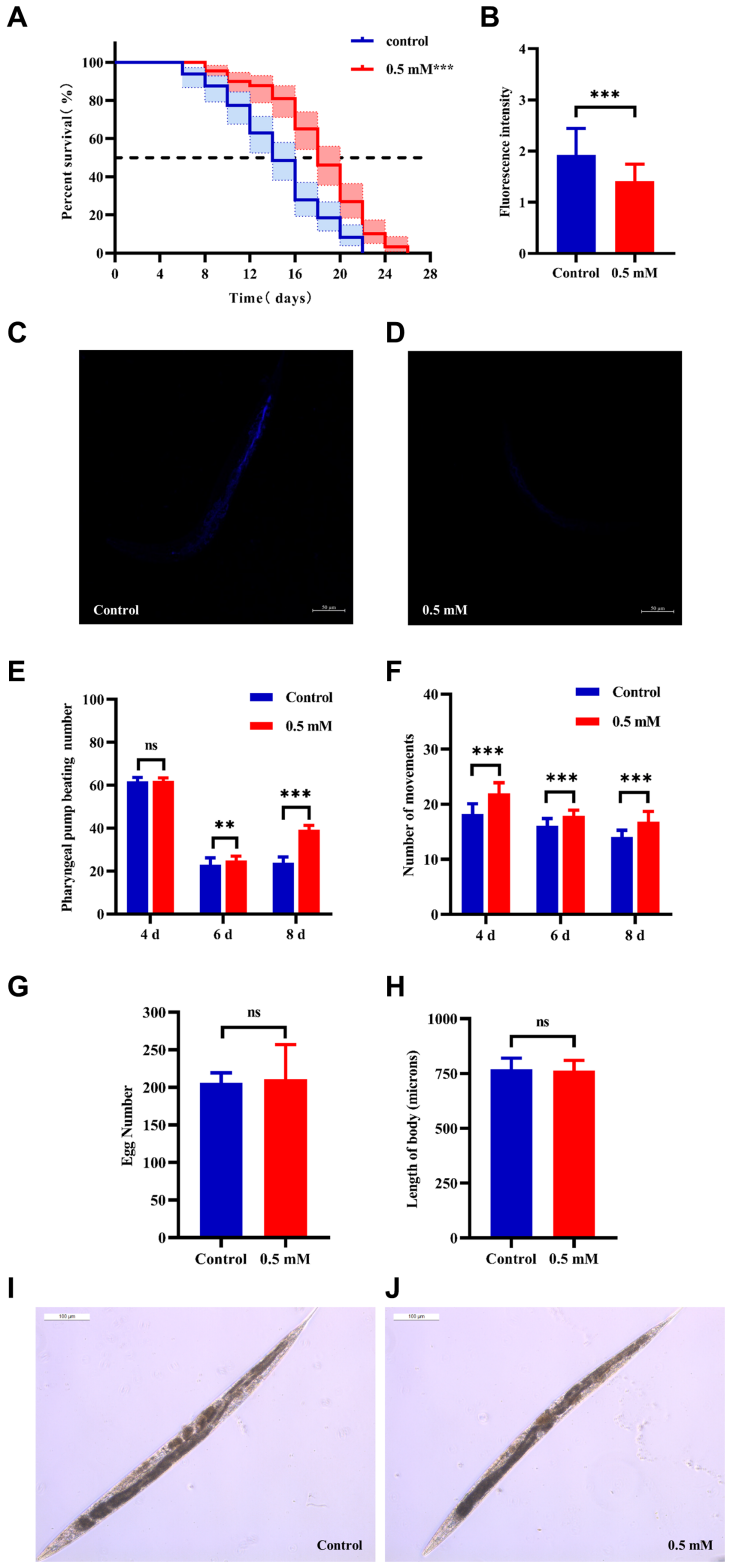
Effects of *p*-HBA on senescence, motility and growth and development of worms. **(A)** Effect of *p*-HBA on lifespan of AD transgenic CL4176 worms: CL4176 worms were treated with 0 mM (blank control) and 0.5 mM *p*-HBA. Treatment prolonged the median and mean lifespan of the worms, and the time to half-survival was also significantly prolonged (*p* < 0.01). The experiment was repeated three times independently. Lipofuscin levels were examined in worms: **(B)** Analysis of lipofuscin fluorescence intensity (*p* < 0.001). **(C)** Representative fluorescence images of the control group. **(D)** Representative images of 0.5 mM treatment group. *p*-HBA on AD worm motility study: **(E)** Frequency of pharyngeal pump beating of worms within 30 s on days 4, 6, and 8. **(F)** Treatment and observation times were the same as in the pharyngeal pump beating experiment and the frequency of sinusoidal movements of worms within 30 s on days 4, 6, and 8. *p*-HBA effects on worm growth and development: **(G)** Analysis of the total number of eggs laid in the control and 0.5 mM groups (*p* > 0.05). **(H)** Worm body length was analyzed using Image J software measurements (*p* > 0.05). **(I)** Representative images of worm body length in the control group. **(J)** Representative images of worm body length in the 0.5 mM treatment group. ^*^p < 0.05, ^**^p < 0.01, ^***^p < 0.001.

Aging plays an important role in the development of AD (Rani et al., 2023). The CL4176 worms were synchronized and cultured and then treated with drugs. Worm survival was observed at intervals, and the results showed that compared to the control group, the survival curve of the worms in the *p*-HBA group was changed to the right (Figure 2A), and the maximum life span increased by 18.182%, and the median life span was 12.461 ± 0.453 days and 16.173 ± 1.081 days, respectively; the mean life spans were 14.454 ± 0.518 days and 18.100 ± 1.036 days, respectively, with an average life extension of 25.225% (Table 3, *p* < 0.001). In addition, lipofuscin is closely related to the aging of worms because it continuously accumulates in the intestine of worms as they age (Höhn et al., 2010). However, our study showed a significant decrease in lipofuscin content in worms after drug administration (Figures 2B–D, *P* < 0.001). These data suggest that *p*-hydroxybenzaldehyde may delay worm senescence.

**Table 3:** Experimental statistics of CL4176 worm lifespan (*x* ± *s*, *n* = 3).

Groups	Maximum lifespan/days	Median lifespan/days	Life expectancy/days	Average life extension rate/%
Control	22	12.461 ± 0.453	14.454 ± 0.518	—
*p*-HBA 0.5 mM	26	16.173 ± 1.081^**^	18.100 ± 1.036^**^	25.225

**p < 0.01.

Besides, with age, the ability of the worms to pump their pharynx decreased, and increasing the frequency of pumping suggests that the drug delayed worm aging. See Figure 2E, compared with the control group, there was no significant difference in the pharyngeal pump beating frequency of the worms in 30 s at day 4 after drug administration (*p* > 0.05), however, at day 6 and day 8, the pharyngeal pump beating frequency in 30 s was increased in both control and drug-administered worms. This result indicated that the drug significantly increased the frequency of pharyngeal pumping of worms at d 6 and d 8 (*p* < 0.001). In addition to this, worm motility also has a strong correlation with longevity, and the measurement of this index responds to whether the drug enhances worm vigor to delay aging. In this study, we investigated the effect of 0.5 mM *p*-HBA on the motility of worms. See Figure 2F, compared to the Control group, worm motility improved at days 4, 6, and 8 after drug administration (*p* < 0.001). There is a direct correlation between the decline in reproductive function and the aging of the life span (Shen et al., 2021). The results of the experiment showed that the total number of eggs laid by the worms in the control group and the administered group was 205.9 ± 13.52 and 210.8 ± 46.12, respectively, and the difference between the two groups was not statistically significant (*p* > 0.05), which indicated that the drug was not reproductively toxic to the worms and had no effect on their reproductive ability (Figure 2G). Next, the worm body length assay showed no statistically significant difference in worm body length after drug administration compared to the control group (*p* > 0.05). This indicated that there was no effect on worm development after drug administration (Figures 2H–J).

### 3.3 *p*-HBA enhances stress resistance and reduces ROS levels in N2 worms

**Figure 3:**
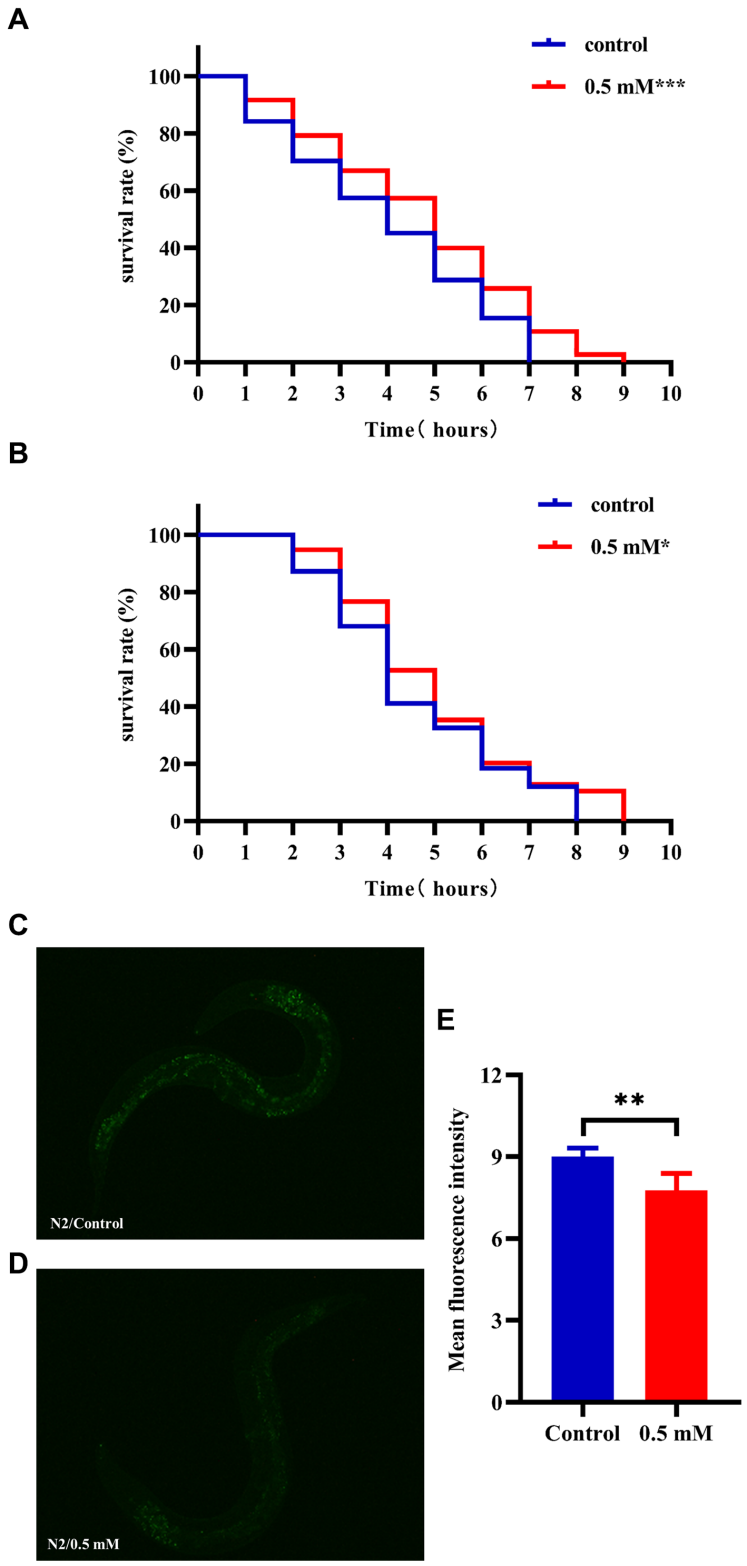
Effect of *p*-HBA on stress resistance and *in vivo* ROS levels in N2 worms. 0.5 mM *p*-HBA treatment improves the resistance of worms: **(A)** Survival curves of worms under high temperature stress at 37°C. **(B)** Survival curves of worms in Juglone (300 μM) constructs for oxidative stress damage environment were analyzed. The experiment was independently repeated three times. Moreover, *p*-HBA reduced ROS levels in N2 worms. Paraquat (5 mM) was used to induce ROS production in worms, and pictures were taken under a fluorescence microscope after staining with DCFH – DA solution. **(C)** Representative pictures of N2 worms without *p*-HBA treatment. **(D)** Pictures of 0.5 mM *p*-HBA-treated N2 worms. **(E)** Fluorescence intensity was measured and analyzed using Image J software. ^*^p < 0.05, ^**^p < 0.01, ^***^p < 0.001.

High temperatures lead to internal metabolic disorders and enzyme inactivation in the organism, which in turn generated large amounts of ROS causing oxidative stress damage (Li et al., 2021). We investigated changes in the resistance to heat stress of N2 worms after the intervention of drug administration at 37°C. After 7 h, all the worms in the Control group died and the survival rate of worms in the *p*-HBA group was 10.811%. As shown in Figure 3A, treatment with 0.5 mM *p*-HBA prolonged the time of worm death and improved the overall worm lifespan (*p* < 0.001). The results suggest that *p*-HBA improved the ability of worms to resist heat stress injury. Administration of *p*-HBA reduced oxidative stress in worms. Next, juglone (300 μM) was chosen as a strong oxidant to construct an environment of oxidative damage, and the ability of *p*-HBA to enhance the antioxidant damage ability of the worms was tested by observing the survival of the N2 worms. As shown in Figure 3B, compared with the control group, the survival curve of the worms in the 0.5 mM *p*-HBA group was shifted to the right and the overall survival time was prolonged (*p* < 0.05). It indicated that *p*-HBA helped improve the ability of worms to resist damage from oxidative stress.

The ROS free radicals are produced when the organism is subjected to oxidative damage, and the excessive accumulation of ROS free radicals will cause an imbalance in the oxidative system, resulting in the organism being in a state of oxidative stress, further promoting damage caused by oxidative stress. The level of ROS in the worms was quantified by fluorescence after DCFH-DA staining, and the higher the amount of green fluorescence, the greater the accumulation of ROS. Paraquat was used to induce an increase in free radicals in worms. See Figure 3E, the mean fluorescence intensities of the two groups were 9.007 ± 0.311 and 7.775 ± 0.612, respectively (*p* < 0.01). Compared with the Control group, the green fluorescence of the nematodes in the *p*-HBA group was weakened (Figures 3C,D), and the ROS level per unit area was significantly reduced (*p* < 0.01). The results indicated that *p*-HBA could inhibit the production of free radicals in nematodes and improve the antioxidant capacity of worms.

### 3.4 *p*-HBA reduced Aβ deposition fiber levels in AD worms

**Figure 4:**
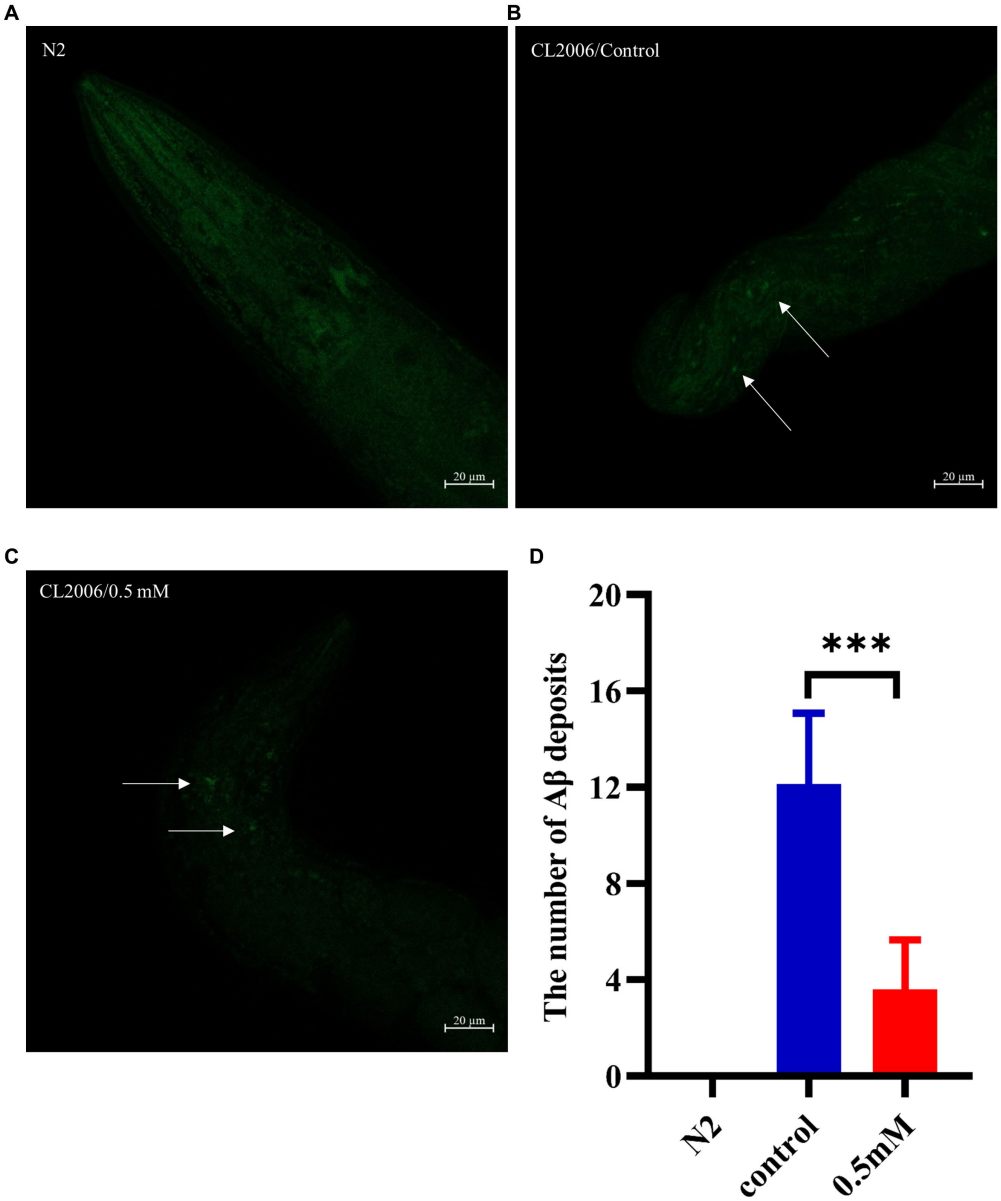
Determination of the number of Aβ protein deposits by thioflavin S staining in transgenic worms CL2006. In CL2006 worms, Aβ protein stained with thioflavin S produces distinct fluorescent patches near the pharynx, with white arrows indicating Aβ deposition sites. **(A)** Wild-type N2 worms served as a negative control with no Aβ protein deposition. **(B)** Representative images of CL2006 worms not treated with *p*-HBA, with more deposits. **(C)** Representative images of worms under 0.5 mM *p*-HBA treatment with significantly less deposits. **(D)** Number of Aβ protein deposits in each group ***p < 0.001.

Aβ formation is an important factor in the pathogenesis of AD, and effective prevention of Aβ formation and increase is important for the treatment of AD (Zhang et al., 2021). In this study, we used the fluorescent dye thioflavin S stain to observe the formation of Aβ deposition fibers and determine the effect of *p*-HBA on Aβ protein aggregation in worms. The experiments were conducted using transgenic CL2006 worms, a worm strain that expresses Aβ protein fragments in muscle cells associated with the development of AD, leading to progressive paralysis. As shown in Figures 4A–C, fluorescence imaging of the head of the worms showed that there was no Aβ deposition in wild-type N2 worms and the administration of the drug significantly reduced the amount of Aβ deposition in the worms compared to the control group (*p* < 0.001). The results suggest that *p*-HBA inhibited the aggregation and deposition of Aβ in muscle cells of transgenic CL2006 worms, thus delaying worm paralysis (Figure 4D).

### 3.5 RNA-seq analysis and validation of *p*-HBA-treated AD worms

**Figure 5:**
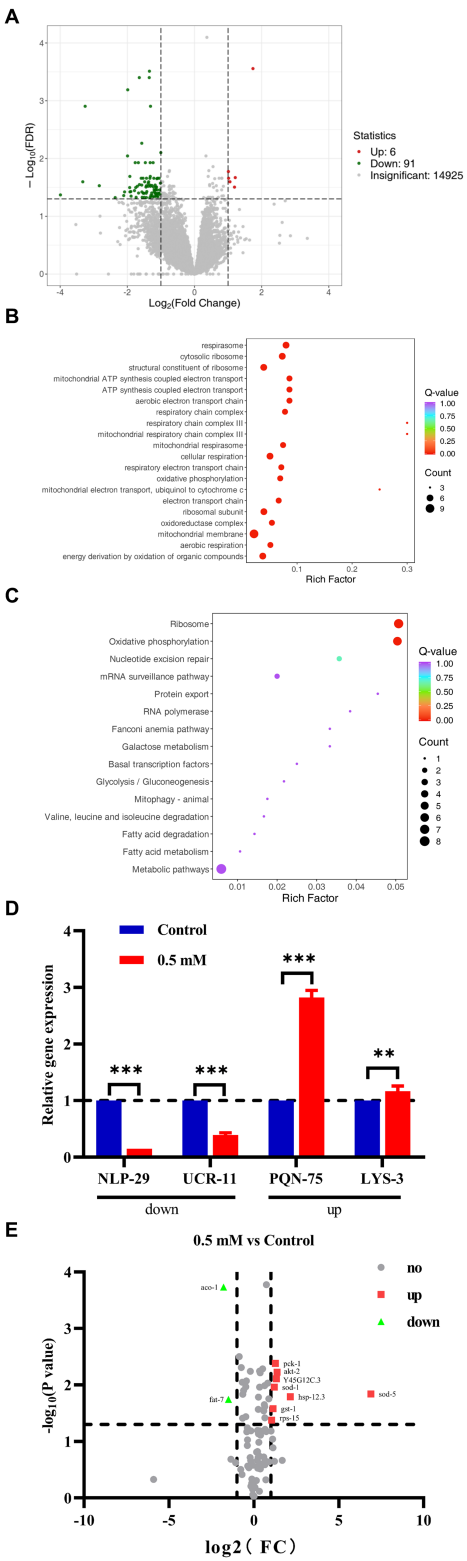
We performed RNA-seq analysis and qPCR validation of the AD worm CL4176. Worms of CL4176 synchronized to L1 stage were transferred to NGM plates with or without drug (0.5 mM), incubated at 16°C for 48 h, then warmed up to 25°C and continued to be incubated for about 30 h. Worms were collected in EP tubes with M9 buffer and washed 2–3 times with sterile water. Sequencing analysis was performed after snap-freezing with liquid nitrogen. **(A)** Differential genes between groups, and the number of up- and down-regulations. **(B)** Differential pathways analyzed by GO enrichment. **(C)** The 15 pathways identified by KEGG enrichment analysis. **(D)** Based on the results of RNA-seq analysis, qPCR analysis was performed to validate four genes related to stress response. The expression trend of these genes was the same as that of RNA-seq analysis, and the mechanism of *p*-HBA anti-AD may be related to its anti-stress effect. **p < 0.01, ***p < 0.001 Subsequently, PCR Array was used for analytical validation of AD-related genes and pathways. Sample preparation was consistent with RNA-seq analysis experiments. Then RNA was extracted and reverse transcribed into cDNA, which was operated according to the instructions of the Gene Chip kit, and the data were exported for analysis. **(E)** Volcano plots reflecting differential genes and expression trends between groups. A total of 10 differentially expressed genes were screened with |log2Fold Change| > =1 and *p* < 0.05 as the condition, including two genes with down-regulated expression and eight genes with up-regulated expression, which were related to AD, aging/anti-aging, ribosomal and glucose metabolism pathways, respectively.

RNA-Seq analysis: with |log2Fold Change| ≥1 and FDR < 0.05 was set to screen for differentially expressed genes (DEGs). A total of 97 DEGs were identified, including 91 down-regulated genes and 6 up-regulated genes (Figure 5A). The biological process was evaluated using Gene Ontology (GO) enrichment analysis, which indicated that the regulatory mechanism of *p*-HBA was related to the “cytoplasmic ribosomes,” “oxidative phosphorylation,” “mitochondrial ATP synthesis coupled with electron transport,” and “respiratory chain complex” pathways (Figure 5B). Furthermore, a total of 15 pathways were identified by KEGG enrichment analysis, e.g., pathways related to “ribosome,” “oxidative phosphorylation,” “Glycolysis/Gluconeogenesis,” and “metabolic pathway” (Figure 5C).

To verify the results of RNA-seq, qPCR analysis was performed. Four genes associated with the stress responses, including hypoxia, hyperosmotic response, temperature change, and heavy metal ion-induced stress, were used for validation (Supplementary Figure>Supplementary Table S2), and these gene regulatory processes are believed to be associated with oxidative stress processes, which in turn can be involved in the regulation of aging-associated disorders such as those that affect the pathogenesis and course of AD. As shown in Figure 5D, the expression of the NLP-29 and UCR-11 genes was negatively regulated, and the expression of the PQN-75 and LYS-3 genes was positively regulated compared to the Control group (^**^p < 0.01; ^***^p < 0.001). These four genes showed the same trend as that observed in the RNA-seq experiments. Thus, the regulatory mechanism by *p*-HBA might be related to the anti-stress response based on the functions and characteristics of these genes.

### 3.6 PCR array analysis of *p*-HBA-treated AD worms

Based on the KEGG enrichment results in the RNA-seq analysis, gene chips (wc-mRNA0612-C, Shanghai Woji Gene Technology Co., Ltd.) related to AD, aging/anti-aging, ribosomal and glucose metabolism pathways were used to explore differences in different signaling pathways or key genes related to AD. Results of the PCR array analysis: the results were analyzed with |log2Fold Change| ≥1 and p < 0 05 as screening conditions. The heatmap can reflect the relative expression multiplicity of samples (Supplementary Figure>Supplementary Figure S1). A total of 10 differentially expressed genes were screened, including 2 genes with down-regulated expression and 8 genes with up-regulated expression, i.e., the expression of ACO-1 and FAT-7 genes was down-regulated, and the expression of AKT-2, GST-1, HSP-12.3, PCK-1, RPS-15, SOD-1, SOD-5, and Y45G12C.3 genes was up-regulated (Figure 5E).

### 3.7 *p*-HBA influenced the expression of DAF-16/FOXO, SKN-1/NRF2, GST-4 and SOD-3 in worms

**Figure 6:**
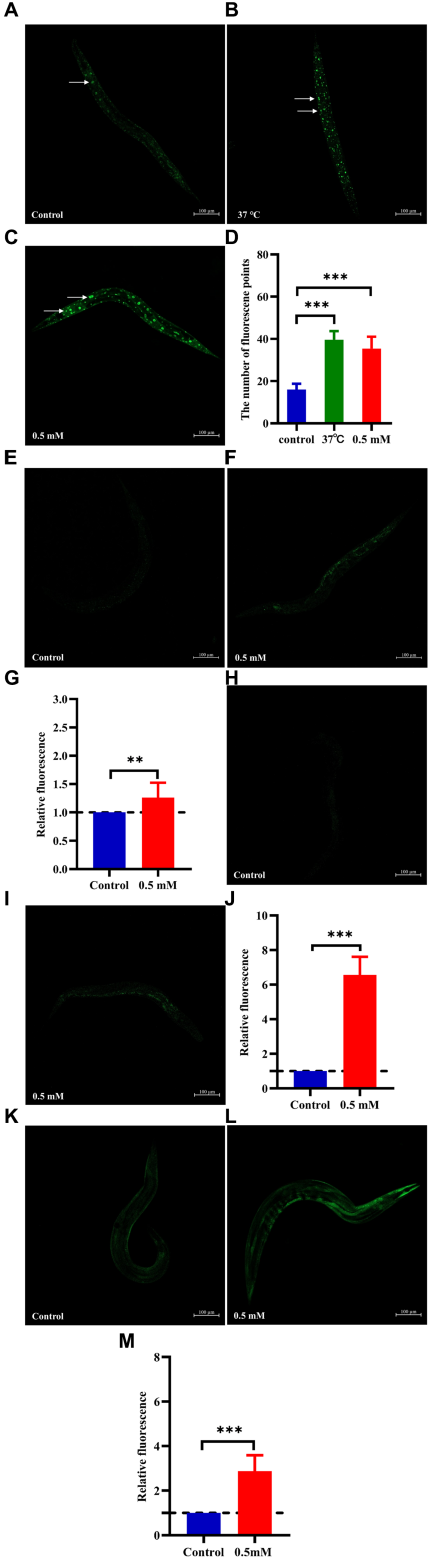
Effect of *p*-HBA on the expression of DAF-16::GFP, SKN-1::GFP, SOD-3::GFP and GST-4::GFP in several reporter-gene worms. TJ356 worms: fluorescence images were obtained under a laser confocal microscope, and green fluorescent spots indicated the nucleus localization of DAF-16 in TJ356 worms (with white arrows to indicate the loci), treated at 37°C for 30 min as a positive control. **(A)** Control group. **(B)** Positive control group. **(C)** 0.5 mM *p*-HBA group. **(D)** Number of fluorescence points in each group (*p* < 0.001). LD1 worms: **(E)** Control group. **(F)** 0.5 mM *p*-HBA group. **(G)** Relative fluorescence intensity measurements of SKN-1::GFP in LD1 worms (*p* < 0.01). CF1553 worms: **(H)** Control group. **(I)** 0.5 mM *p*-HBA group. **(J)** Fluorescence intensity expression analysis of SOD-3::GFP in worms (*p* < 0.001). CL2166 worms: **(K)** Control group. **(L)** 0.5 mM *p*-HBA group. **(M)** Relative fluorescence intensity of GST-4::GFP expression in CL2166 worms (*p* < 0.001). Experiments were independently repeated 3 times.

The nuclear localization of DAF-16 and SKN-1 was determined using the strains TJ356 and LD1, respectively. Treatment with *p*-HBA accelerated the nuclear translocation of DAF-16 (*p* < 0.001) (Figures 6A–D). Also, *p*-HBA treatment relatively enhanced SKN-1 expression in LD1 worms (Figures 6E–G), and thus, it was hypothesized that the inhibitory effect of *p*-HBA on Aβ toxicity and enhanced antioxidant properties was due in part to the activation of DAF-16. To clarify the molecular response to the protective effects of *p*-HBA in *C. elegans*, we examined the expression of two genes associated with longevity and resistance to oxidative stress, including SOD-3 and GST-4. SOD-3 is involved in the protection of *C. elegans* against oxidative stress and inhibits the toxicity of Aβ (Cao et al., 2022). Glutathione transferase can play a role in the detoxification of oxidative stress products, and GST-4, as a bifunctional enzyme in the σ class of the glutathione transferase family, not only participates in the glutathione metabolism process, but also enhances the activity of glutathione transferase (Chen et al., 2020). *p*-HBA treatment enhanced the fluorescence intensity of SOD-3::GFP in the CF1553 strain *in vivo* (Figures 6H–J), and the fluorescence of the CL2166 strain that expresses GST-4:: GFP was also significantly increased by *p*-HBA treatment (Figures 6K–M), suggesting that *p*-HBA enhances the expression of SOD-3 and GST-4 in worms (*p* < 0.001), which could delay paralysis and prolong the lifespan of worms by activating SOD-3 and GST-4.

## 4 Discussion

In recent years, as human living standards continue to improve and the aging of the world’s population rises dramatically, aging-related neurodegenerative diseases such as AD have received increasing attention and focus. AD, which accounts for the highest proportion of dementia-like diseases, is an extremely insidious progressive neurodegenerative disease with clinical symptoms including memory impairment, aphasia, and other manifestations of dementia (Liu et al., 2022). Many of its induced complications also lead to a large number of patient deaths. Currently, the number of patients with AD is increasing year by year, which has become a very serious social and public health concern (Muñoz-Montero et al., 2022). We already know that existing drugs cannot meet the needs of the society (Ju and Tam, 2022), so prevention and treatment of AD disease and the development of related drugs are urgent. Regarding the pathogenesis of AD, the main hypothesis is the *β*-amyloid cascade hypothesis, which suggests that the aggregation of Aβ in the brain is the main predisposing factor in AD. And oxidative stress occurs in the early stages of AD pathology, when the organism is exposed to a variety of harmful stimuli, the excessive production of ROS in the body leads to an imbalance in the oxidative and antioxidant systems (Shukla et al., 2011), which not only affects the mitochondrial energy metabolism, accelerates the phosphorylation of tau proteins (Sierra-Fonseca and Gosselink, 2018) and plays a role in neurotoxicity through the formation of neurofibrillary tangles and lowering of the excitability threshold, but also it also causes hyperoxidation of functional cell membrane proteins and lipids, destabilizing the cell membrane bilayer and contributing to the accumulation of Aβ protein, which in turn accelerates the generation of reactive oxygen species, induces neuronal cell damage and death, and leads to the occurrence of AD (Alafuzoff et al., 2008; Mohsenzadegan and Mirshafiey, 2012). At the same time, many studies have shown (Reddy et al., 2016; Feng et al., 2019; Zhou et al., 2019; Song et al., 2022) that effective inhibition of Aβ aggregation and improvement of the body’s antioxidant level are important tools in the prevention and treatment of AD.

Currently, several model animals are available to evaluate treatment effects and to perform mechanistic studies of anti-AD drugs, including mice, dogs, and non-human primate models. However, they all have limitations, such as a long experimental period, high costs, and ethical issues. Therefore, in this study, we used the model organism *C. elegans*. Compared with other model animals, *C. elegans* has the advantages of a short life cycle, small size, relatively low experimental cost, it is easy to cultivate and observe, and has a high degree of homology with human genes (Shaye and Greenwald, 2011; Shen et al., 2018). Furthermore, scientists have established many readily available *C. elegans* models for AD, such as the CL2006, CL4176, and CL2166 strains of nematodes (Fay et al., 1998; Kampkötter et al., 2007). In recent years, researchers have made good progress using these transgenic *C. elegans* models to conduct a number of AD-related studies. For example, *Lippia origanoides* essential oil (LOEO) (Henrique Moniz et al., 2023), Hesperidin (Kumar et al., 2023), A fruit extract of *Styphnolobium japonicum* (L.) (Thabit et al., 2023) have also been found to be efficacious in the treatment of AD in nematode model-based studies. Overall, the practice of AD drug development using the *C. elegans* model has become more mature, providing a bright prospect for high-throughput screening of drugs for neurodegenerative diseases (Paul et al., 2020).

In this study, we used one of the major phenolic components of the EEGE site, *p*-HBA, which is a phenolic aldehyde and also an isomer of hydroxybenzaldehyde (Loh et al., 2022). As a major intermediate in the spice and pharmaceutical industries, it is an important organic compound for the production of flavonoid precursors (Bountagkidou et al., 2010; Loh et al., 2022). In addition, as one of the main active components of *Gastrodia elata* (He et al., 2016), studies have demonstrated its potent pharmacological effects as an anti-inflammatory (Bin et al., 2017), anti-oxidative stress injury (Xiao-nan et al., 2017), anti-angiogenic and neuroprotective agent (Jung et al., 2007; Kim et al., 2007). We used the transgenic AD CL4176 worms as a model system. The model induces Aβ toxicity to investigate the protective effects of *p*-HBA against Aβ proteotoxicity *in vivo*. *p*-HBA delayed Aβ-induced paralysis and prolonged the general paralysis of the worms, but the lower and higher administered did not produce an effect, which may be related to the *in vivo* metabolic pattern of *p*-HBA (Jin et al., 2020; DanQing et al., 2021; Long et al., 2021). By evaluating half the paralysis time and the overall paralysis time (Keowkase et al., 2018), we determined that the optimal effective concentration of *p*-HBA was 0.5 mM and we used this concentration for subsequent studies. First, we performed reproductive, lifespan, and behavioral studies using the CL4176 worm model, including measurements of pharyngeal pump beating and motility. *p*-HBA did not exert any effects on AD worm reproduction but prolonged the lifespan of AD worms by enhancing worm motility during drug administration, which in turn slowed the aging process. At the same time, it can reduce the amount of lipofuscin in the intestinal tract of worms, suggesting that it has an anti-aging effect. It also had no effect on worm development. Oxidative stress plays a key role in the pathological course of AD (Mohamed et al., 2021). In addition, high temperature induces a homeostatic imbalance in the organism and excessive ROS production, causing oxidative stress. In this study, we investigated the role of *p*-HBA to evaluate the response to heat stress in the organism using a wild-type N2 worm model. The results showed that it could prolong the survival time of N2 worms under heat stress conditions and improve their resistance to heat stress damage. Second, we used Juglone to construct an oxidative stress model to investigate whether *p*-HBA could enhance the antioxidant capacity of wild-type N2 worms. Unsurprisingly, *p*-HBA did enhance the ability of the worms to resist oxidative stress injury. Based on the antioxidant properties of *p*-HBA, we measured the accumulation of ROS in the worms, and the administration of *p*-HBA reduced the average fluorescence intensity of the whole worms and reduced ROS production compared with controls. As Aβ protein is one of the main parameters in the study of AD pathology (Paul et al., 2020), we confirmed that *p*-HBA reduced the aggregation of Aβ protein in the transgenic worm strain CL2006 using thioflavin S staining, which in turn delayed the paralysis of the worms. The above results suggest that *p*-HBA plays an important role in antioxidant, anti-aging, anti-Aβ aggregation and antitoxic activity.

RNA-seq sequencing analysis was used to explore the target and mechanism of action of *p*-HBA to improve AD-like symptoms in transgenic *C. elegans* at the gene level. We screened *C. elegans* genes and genes involved in signaling pathways related to Aβ toxicity and oxidative stress, and verified DEGs by RT-PCR, to further investigate the mechanism of action underlying *p*-HBA anti-AD effects. Through gene function analysis, four genes that may be related to the pathological process of AD were identified. They were NLP-29, UCR-11, PQN-75, and LYS-3, which were associated with the stress response. RT-qPCR showed that the relative expression trends of these genes were consistent with the RNA-seq findings. Among them, NLP-29 belongs to antimicrobial peptide genes, which induce cell repair in response to epidermal damage and are associated with innate immunity (Pujol et al., 2008; E et al., 2018; Chandler and Choe, 2022), and is expressed at low levels in adult worms under normal conditions and is up-regulated when exposed to osmotic stress. Conversely, the gene will be relatively unexpressed or down-regulated when the drug enhances resistance to hyperosmotic stress injury, which is consistent with our findings and suggests that *p*-HBA has a protective effect against hyperosmotic stress injury. Mitochondrial protein homeostasis is closely related to aging and many diseases associated with aging, including AD (Ross and Poirier, 2004; Opattova et al., 2013). UCR-11 is a complex III electron transport chain protein involved in mitochondrial electron transport, and studies have shown that this gene forms extensive aggregates in mitochondria after hypoxia, but cytoplasmic protein aggregation is a hallmark of aging (Labbadia and Morimoto, 2015); researchers have demonstrated that this aggregation of mitochondrial proteins is part of the senescence process. However, when resisting the hypoxic stress response, the relative expression of this protein is reduced, thus slowing the aging process (Kaufman et al., 2017), which is similar to our findings, and suggests that *p*-HBA can slow down the aging process by resisting hypoxic stress. PQN-75 is expressed in pharyngeal gland cells of *C. elegans*, and studies have shown that it protects the organism from acute temperature changes and has a protective effect on the high-temperature-induced stress response (Rochester et al., 2017). Thus, the up-regulation of PQN-75 expression after administration of *p*-HBA suggests that *p*-HBA helps to resist high-temperature stress injury. Furthermore, the gene review revealed that LYS-3 is involved in the response to copper ions stress by exerting a certain protective and defensive effect (Hattori et al., 2013), and its up-regulated expression suggests that the drug has a certain protective effect on the response to heavy metal ion stress. In fact, stress indicates the resistance capacity of the organism in stressful environments, and there is a strong correlation between the extension of lifespan and the improvement of the ability to pressure-resistant. As shown above, *p*-HBA has a protective effect on stress responses, including hypoxia, hyperosmotic response, acute temperature, and heavy-metal-induced stress, which can further delay aging and resist aging-associated diseases. Secondly, we analyzed the more critical pathways such as ribosomes and oxidative phosphorylation pathways by KEGG and GO enrichment of differential genes. Ribosomes are known to play an important role in the aetiology of AD, and their structural and functional stability is closely associated with neurodegenerative diseases (Li et al., 2018; Nguyen et al., 2024). Existing studies have shown that increased ribosome function is more common in patients with AD, and inhibition of ribosome biosynthesis may be associated with the efficacy of pharmacological treatments for this disease (Suzuki et al., 2022; Feng et al., 2024). Our results showed downregulation of relevant ribosomal genes after drug administration, which is consistent with previous studies (Feng et al., 2024), suggesting that targeted modulation of the ribosomal pathway and related genes is potentially relevant for the treatment of Aβ-induced AD. In addition, the oxidative phosphorylation pathway is also closely linked to the developmental process of neurodegenerative diseases such as AD. Mitochondria provide most of the energy for the organism through the oxidative phosphorylation pathway, which in turn is involved in a variety of physiological and biochemical processes such as cellular signaling, inflammatory response and apoptosis (Chuanzhu et al., 2020). In contrast, dysfunction of mitochondrial oxidative phosphorylation induces neurodegenerative disease processes. Studies have shown that a natural hallucinogen, *N*, *N*-dimethyltryptamine, contributes to the amelioration of AD by modulating oxidative phosphorylation in an *in vitro* model of AD (Cheng et al., 2024), so similarly *p*-HBA may play a role in ameliorating the AD *C. elegans* model by mediating the oxidative phosphorylation pathway.

Next, we validated the expression of AD-related pathways using customized gene chips. A total of 10 DEGs were identified using |log2Fold Change| ≥1 and *p* < 0.05 as the screening condition. These DEGs were related to AD, aging/anti-aging, glucose metabolism and ribosomal pathways, respectively. Among the genes related to AD: the expression of FAT-7, SOD-1, SOD-5, GST-1, and HSP-12.3 was up-regulated. FAT-7 is involved in fatty acid biosynthesis (Shi H. et al., 2023), SOD-1 and SOD-5 are both members of the superoxide dismutase family, and SOD is directly involved in the removal of superoxide radicals, which can regulate the levels of cell-produced superoxide and hydrogen peroxide, which in turn regulates cell signaling (Brieger et al., 2012). GST-1 is a member of the glutathione transferase family, and GST is mainly involved in the inhibition of oxidative stress, which protects the organism from damage by oxidative stress (Urbizo-Reyes et al., 2022). HSP-12.3 belongs to the heat shock protein (HSP) family, and small HSPs can improve survival of nematode under stress (Webster et al., 2019), enabling HSP binding activity, maintaining cytoskeletal stability, and therefore promoting cell survival, which is an important regulator of cell growth and differentiation (Yang et al., 2020). The above suggests that *p*-HBA treatment reduces fat accumulation, enhances the expression of antioxidant genes, and thereby exerts antioxidant and anti-AD effects by protecting cells from redox damage. ACO-1 expression was down-regulated and AKT-2 and PCK-1 expression was up-regulated. These are genes related to glucose metabolism. ACO-1 is involved in the tricarboxylic acid cycle process, and it has been shown that ACO-1 up-regulation responds to *Shigella flexneri* to disrupt iron homeostasis of infected cells and also induces hypoxia response leading to worms death; however, silencing of ACO-1 expression renders the worms more resistant to the negative effects caused by *Shigella flexneri* (George et al., 2014). AKT activation is a positive regulator of metabolic control, of cell growth and proliferation, angiogenesis, protein synthesis, and neuronal survival (Manning and Cantley, 2007). Some studies have shown that exercise training has the potential for the prevention and treatment of neurodegenerative disorders, possibly due to the fact that exercise stimulates AKT-1 and AKT-2 proteins associated with signaling proteins related to the insulin/IGF-1 pathway, thus exerting a positive effect in improving memory deficits (Diegues et al., 2014). Although PCK-1 is involved in several processes, including carbohydrate biosynthesis processes, cellular responses to oxygenated compounds, and propionate catabolism processes, inhibition of PCK-1 affects glucose metabolism (Elcoroaristizabal Martín et al., 2011). Furthermore, the ribosome-associated protein gene RPS-15 and Y45G12C.3, an aging/anti-aging related gene, were both up-regulated in expression; RPS-15 is located in the ribosome and is a ribosomal component involved in determining adult lifespan, and Y45G12C.3 is involved in glutathione metabolism processes, which are affected by genes related to the insulin pathway such as DAF-2 and DAF-16. From the above, it can be seen that *p*-HBA treatment has a certain positive effect on genes related to AD pathways, glucose metabolism, ribosomal protein pathway and anti-aging, and work together to participate in the protective mechanism of *p*-HBA against AD.

The insulin signaling pathway is an important lifespan regulation pathway, DAF-16 is an important swim transcription factor of the insulin IIS pathway, DAF-16 is expressed in large amounts, which can delay aging and prolong the lifespan of worms, and SKN-1, GST-4, and SOD-3, are the target genes of DAF-16 (FOXO transcription factor homolog DAF-16), which can be used to inhibit AD development by catalyzing the removal of O^2−^ to protect the organism from oxidative stress, thereby inhibiting the process of AD development (Wang et al., 2016; Tullet et al., 2017). We used *C. elegans* strains TJ356 (DAF-16::GFP), LD1 (SKN-1::GFP), CF1553 (SOD-3::GFP), and CL2166 (GST-4::GFP) to examine the molecular mechanism of *p*-HBA. It not only affected the nuclear translocation of DAF-16, but also enhanced the expression of SKN-1 in LD1 worms. Nonetheless, *p*-HBA enhanced the expression of SOD-3 in strain CF1553. SOD-3 is located in the mitochondrial respiratory body and is expressed in several structures and can enable proteins with homodimerization activity and superoxide dismutase activity, scavenging superoxide radicals, and playing an important role in resisting oxidative stress processes. We also observed that the fluorescent expression of the transgenic CL2166 worms increased with *p*-HBA treatment, suggesting that upregulation of GST-4 gene expression by *p*-HBA also contributes to protection against Aβ-induced toxicity in AD worms.

## 5 Conclusion

This study found for the first time that *p*-HBA, a major phenolic component in *Gastrodia elata*, has a protective effect against *β*-like amyloid toxicity in the *C. elegans* model of AD, which may be achieved through enhanced expression of DAF-16, SKN-1 and GST-4, which in turn reduces endogenous ROS production and increases resistance to stress, and can interact with each other to participate in the protective mechanism of Aβ proteotoxicity. Meanwhile, the nuclear location of DAF-16 promoted the expression of SOD-3, which greatly contributed to the enhancement of motility, reduction of lipofuscin and ROS levels, and prolongation of paralysis time and lifespan of *C. elegans* treated with *p*-HBA. The present study confirmed the antioxidant and anti-aging activity of *p*-HBA and the inhibition of Aβ protein aggregation against toxicity. The above studies provide new perspectives on the potential of *Gastrodia elata* and its monomeric active ingredients in the treatment of patients with AD. For other monomer components of *Gastrodia elata* that have not yet been fully investigated (i.e., Parishin A and Parishin C), our future studies will analyze their therapeutic effects on AD to determine whether the efficacy of *Gastrodia elata* in the treatment of neurodegenerative disorders is holistic, which implies that the application of TCM and ethnomedicine in the treatment of diseases requires a variety of components and targets, rather than a single component and a single target. In addition, we will comprehensively analyze *p*-HBA based on the *C. elegans* model by considering its toxicity and metabolites after treatment in future experiments. However, we also recognize the limitations of using the *C. elegans* AD model. *C. elegans* are invertebrates and lack certain anatomical features of mammals, so there may be some bias in extrapolating the results of this research to human conditions. Therefore, we will continue to investigate the role of *p*-HBA and related molecular mechanisms in different AD animal models to prepare the drug for the next step of clinical trials. In addition, whether *p*-HBA has a protective effect on other neurodegenerative diseases remains to be explored for further research.

## Data availability statement

The datasets presented in this study can be found in online repositories. The names of the repository/repositories and accession number(s) can be found in the article/Supplementary Figure>Supplementary material.

## Author contributions

XY: Conceptualization, Data curation, Formal analysis, Methodology, Software, Validation, Visualization, Writing – original draft. JT: Conceptualization, Methodology, Validation, Visualization, Writing – original draft. TX: Conceptualization, Methodology, Software, Validation, Visualization, Writing – original draft. XD: Conceptualization, Data curation, Funding acquisition, Investigation, Project administration, Resources, Supervision, Writing – original draft, Writing – review & editing.

## Footnotes

^1^https://www.primer3plus.com/
