# Supplementary material for: P-hydroxybenzaldehyde protects Caenorhabditis elegans from oxidative stress and β-amyloid toxicity
Source: Front Aging Neurosci. 2024 May 22;16:1414956. doi: 10.3389/fnagi.2024.1414956 (PMC11150654; doi:10.3389/fnagi.2024.1414956)

Supplementary Material

P-hydroxybenzaldehyde protects *Caenorhabditis Elegans* from oxidative stress and β-amyloid toxicity

Xingzhi Yu^1^, Jie Tao^1^, Tian Xiao^1^, Xiaohua Duan^1*^

^1^Yunnan Key Laboratory of Dai and Yi Medicines, Yunnan University of Chinese Medicine, Kunming, Yunnan 650500, P.R. China

*** Correspondence:**Professor Xiaohua Duan
1047896527@qq.com

**Table 1.** Formulation table for cDNA and mixtures

| Constituent | 96-well (9 µL/well) |
| --- | --- |
| WCGENE^®^ mRNA qPCR mix （2×） | 510 µL |
| cDNA sample | 100 μL |
| RNAase-free ddH2O | 310 μL |
| Overall volume | 920 μL |

**Table 2.** Background information of the four genes

| Gene symbol | Gene Description | Gene function |
| --- | --- | --- |
| Nlp-29 | Neuropeptide-like protein 29 | Antimicrobial peptide gene. Involved in defense responses against other organisms and negatively correlated with anti-hypertonic stress modulation. |
| Ucr-11 | Cytochrome b-c1 complex subunit 10 | A complex III electron transport chain protein. Involved in mitochondrial electron transport, extensive aggregates are formed in mitochondria during aging or hypoxia in the organism and are positively correlated with hypoxic stress. |
| Pqn-75 | Basic proline-rich protein | Expressed in the pharynx, it protects organisms from acute temperature changes. |
| Lys-3 | Lysozyme-like protein-3 | Participates in the stress response to copper ions and protects against heavy metal stress. |

**Figure 1 of the supplementary material**


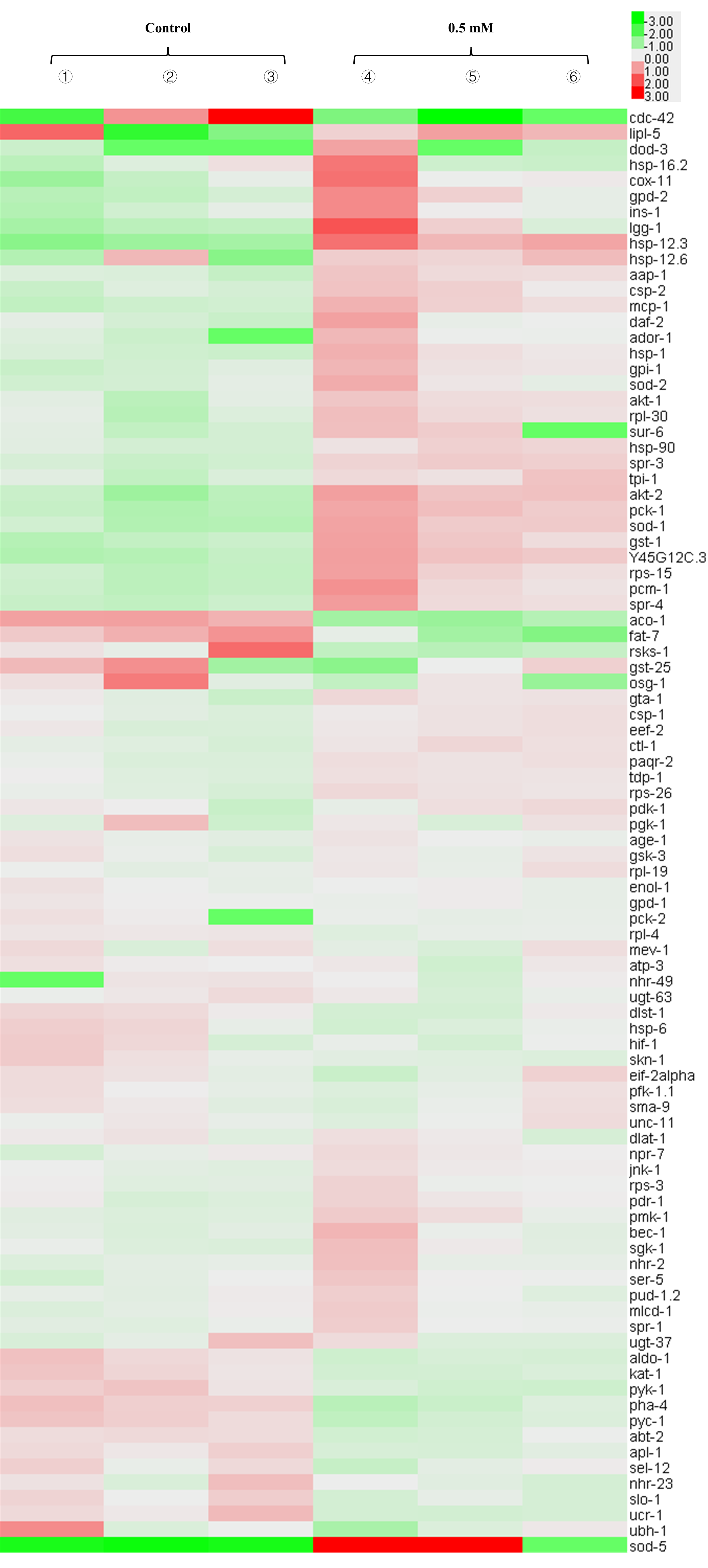


**Supplementary Figure 1.** The heat map reflects the relative multiplicity expression of the two sample groups. Of these, ① ② ③ are Control groups, ④ ⑤ ⑥ are 0.5 mM p-HBA treated groups.

# GSEA Analytical Annotation for KEGG and GO

**NAME**: Gene Set Name;

**GO_ID**: the ID of the GO entry corresponding to the gene set;

**SIZE**: Number of genes contained in the gene set (number greater than 15 and less than 5000);

**ES**: Enrichment Score;

**NES**: Normalized Enrichment Score, which takes into account the size of the gene set and normalizes the ES values for each genome to obtain a normalized enrichment score;

**NOM p-val**: nominal P-value, nominal p-value, statistically significant level for enrichment analysis;

**FDR q-val**: false positive rate q-value, statistically significant level for enrichment analysis obtained after multiple hypothesis testing.

# Raw data upload platform

<https://www.ncbi.nlm.nih.gov/sra/>

<https://submit.ncbi.nlm.nih.gov/subs/sra/>

Submission number: SUB14170595


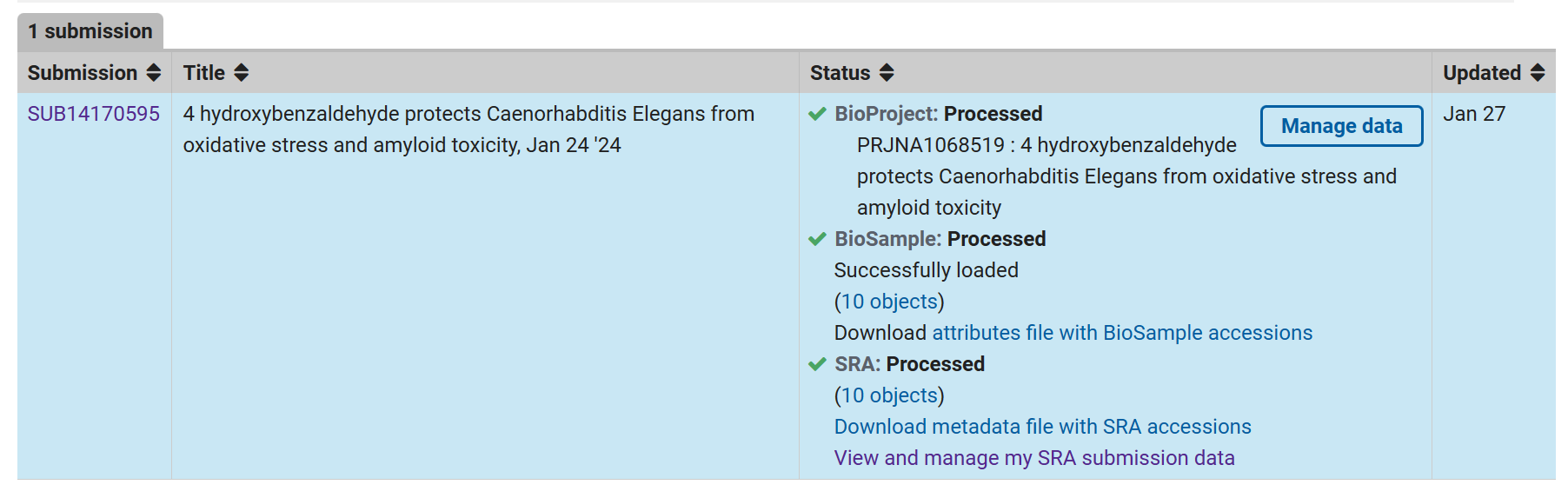

Supplement: Supplementary file 1 [file Data_Sheet_1.docx]
