# Supplementary figures and images for: P-hydroxybenzaldehyde protects Caenorhabditis elegans from oxidative stress and β-amyloid toxicity
Source: Front Aging Neurosci. 2024 May 22;16:1414956. doi: 10.3389/fnagi.2024.1414956 (PMC11150654; doi:10.3389/fnagi.2024.1414956)

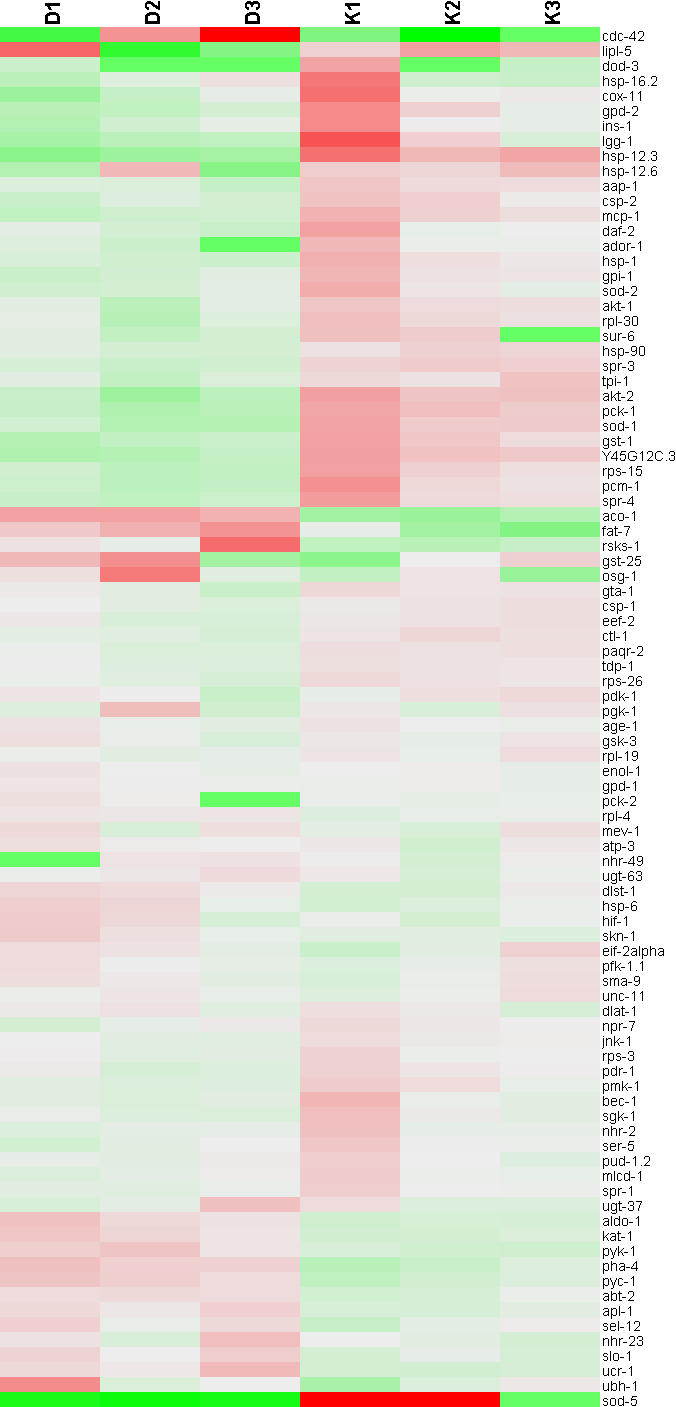

Supplement: Supplementary file 2 [file Data_Sheet_2.zip › PCR Array data/heatmap/heatmap.png]

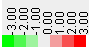

Supplement: Supplementary file 2 [file Data_Sheet_2.zip › PCR Array data/heatmap/heatmap_colorbar.png]
